# Understanding of disease status, prognosis and estimated cost of treatment among cancer patients: experience from a tertiary cancer centre in Nepal

**DOI:** 10.3332/ecancer.2024.1668

**Published:** 2024-02-13

**Authors:** Shweta Baral, Sudhir Raj Silwal, Deep Lamichhane, Abish Adhikari, Nancy Bhattarai

**Affiliations:** Bhaktapur Cancer Hospital, Kathmandu 44800, Nepal; https://orcid.org/0000-0002-6284-1775

**Keywords:** cancer, patients, caregivers, understanding, communication, treatment compliance, Nepal

## Abstract

**Purpose:**

Lack of adequate knowledge about the disease is one of the key factors that misguide cancer patients and patients’ caregivers in choosing a better management plan. The purpose of the study was to assess the patients’ and caregivers' knowledge about the disease status and estimated treatment cost. Understanding of disease may improve adherence to treatment plans and quality of care.

**Methods:**

It was a cross-sectional study where 120 cancer patients were selected based on convenience sampling and the availability of record files and relevant data. The site, stage and prognosis of disease recorded from patients and caregivers were compared with the record of outpatient department and inpatient files with the help of the Fisher's exact test. The patients’ knowledge about their financial estimates was also recorded. Three patients were selected for in-depth interviews based on purposive sampling to further support the findings.

**Results:**

Among 120 analysed patients, 60.83% were female. Around two-thirds of male patients (69.2%) and male caregivers (62.2%) knew about the site but only one-third of female patients (30.8%) and female caregivers (37.8%) knew the same. The primary responsibility for managing financial issues was caregivers in 89.16%. Only 7.5% knew the estimated cost. Nepali as the primary language and better education level is correlated with knowledge of disease status, among both patients and caregivers but was statistically significant only in knowing prognosis among native Nepali speaker caregivers (*p* < 0.001), and better-educated patients (*p *< 0.001). As per the in-depth interview, all three patients knew the site of their disease, but only the patient with breast cancer was aware of the stage of the disease. None of the patients were aware of their disease prognosis, treatment plan or the estimated cost of their treatment.

**Conclusion:**

The level of understanding is low for most patients and their immediate caregivers, particularly among those who are female, not literate and whose primary language is not Nepali. Appropriate strategy should be adopted to enhance basic understanding among patients and caregivers in our setting.

## Introduction

There is a strong disparity in cancer incidence and mortality rates in high-income countries and low-middle-income countries (LMICs) [1–3]. It is estimated that by 2030, approximately three-quarters of all cancer deaths will occur in LMICs, with one in eight people experiencing a cancer diagnosis in their lifetime [4]. Ongoing research studies mostly focus on expanding treatment options ignoring the reasons behind poor treatment outcomes in LMICs.

Lack of adequate knowledge is one of the key factors in LMICs that misguide patients and patients’ caregivers, especially in advanced cancer cases who end up choosing expensive treatment plans leading to financial crisis for themselves without much survival benefits. A study by Chauke *et al* [5], provided evidence that poor medicine adherence for chronic diseases in LMIC is influenced by a lack of knowledge, negative attitudes and negative beliefs leading to poor quality of life. Early-stage cancer patients discontinue their planned treatment not knowing their chance of curability, additionally misguided by the prevalent cancer myths in our society. Moreover, previous studies have demonstrated that knowing the estimated treatment cost helps patients and caretakers make financial plans appropriately, leading to improved treatment compliance [6, 7]. Patient characteristics also contribute to patients’ understanding of their disease course including age, sex, health literacy, cancer stage, cancer type and remission status [8]. In contrast to the Western world, in our population, generally, the patient’s family members are involved in treatment decisions and financial management. In addition to this, the patient’s primary language and educational status may also contribute to the comprehension of the communicated facts [8].

Inadequacy of effective physician–patient communication is also one of the major reasons behind the improper knowledge of disease status among cancer patients. Various studies have proved that good information on disease status and prognosis directs the patient’s preference for a better management plan [9–13].

Furthermore, in our resource-constrained setup, there are other logistic issues like curative full-dose radiotherapy in otherwise palliative intent patients, occupying the radiotherapy slots, increasing the treatment waiting time for the needy patients. In the case of non-curable palliative patients, patients tend to sell their property to meet the treatment expenses in the hope of curing their disease. This, in turn, can cause financial distress for their families, and impact their children's education.

This study evaluated the knowledge of both patients and patients’ caregivers about the disease status. To our knowledge, this type of study is scarce in our population.

## Methodology

### Research design

It was a mixed-method study.

A survey with a semi-structured questionnaire was done with the selected patients and their caregivers about their understanding of disease status and financial estimates. Disease status included the site and stage of the disease. The prognosis was asked in terms of whether they knew the disease was curable or not. Financial estimates refer to knowing the total estimated cost of the disease before starting the treatment.

In-depth interviews were conducted with patients with the aim to further support the findings of the survey. It was ensured that every participant in the interview was allowed sufficient engagement time which facilitated to discover core facts about the issues. The participants were asked about their knowledge of site, stage and prognosis of the disease. Financial queries included the total estimated cost of treatment and the amount they had already spent in different phases of treatment. They were allowed to share their experience about communication with the treating physicians they had during the course of treatment.

### Setting

One hundred and twenty patients were selected for the survey based on convenience sampling who had initiated treatment at various departments of Bhaktapur Cancer Hospital. Patients were selected on the availability of record files and relevant data within a period of 4.5 months from three major departments of the hospital (radiation oncology, medical oncology and surgical oncology), 40 patients from each department with the assumption of the sample being representative of the patients’ population in our setting.

Three patients were selected for in-depth interviews based on purposive sampling to be inclusive to all three major departments of the hospital. Intentionally, patients were chosen from different departments to extract the information specific to the concerned departments. The patients were asked separately about their knowledge of disease status, prognosis, estimated costs and their experiences in the hospital.

### Variables and outcome measures

The demography of patients was recorded from the patients’ outpatient department (OPD) and inpatient files. Variables associated with patients and caregivers were age, sex, education status, their primary language at home and work and the person responsible for managing the cost of treatment. Patients’ and caregivers’ knowledge about their disease site and stage and prognosis were recorded. The site, stage and prognosis recorded from patients and caregivers were then compared with the record of OPD and inpatient files. The patient’s knowledge about their financial estimate was also recorded.

For the in-depth interview, narratives were captured and relevant points with respect to the research purpose were noted down during the interview process. Specifically, patients’ knowledge about site, stage and prognosis of the disease was recorded and their experience of disease and financial communication with physicians were captured. The interviews were also recorded on tape for later analysis.

### Statistical analysis

Cross-tabulation was prepared according to patients' and caregivers’ responses about their disease status, and prognosis compared with the record of the site, stage and prognosis in the clinical record files. Concordance between self-reported cancer sites, stage and prognosis and recorded data were summarised with frequencies and percentages. Fisher’s exact test was used to compare the data and presented in terms of *p*-values. *p*-Value less than 0.05 was considered significant. Analysis was done in IBM SPSS Statistics version 25 software.

For the analysis of in-depth interviews, the recorded audio and interview sheets were re-evaluated, coded and indexed according to the research questions. A deductive approach was used for data analysis for the narrative part. Specific themes were identified and stories were reformulated according to the themes. Conclusions were drawn about the meanings, experiences and perspectives of the patients.

### Ethics statement

Research approval was obtained from the Nepal Health Research Council institutional review board and hospital administration before conducting the study. Informed consent of patients and caregivers was taken before starting the study.

## Results

### Results of quantitative part

One hundred and twenty patients were analysed for the study, with the median age of the patient being 54 years (range:13–79 years). Most of the patients (60.83%) were female. The primary language of about 20% of the participants was not Nepali, 9.16% of them using Newari most frequently in their homes and offices whereas other 9.16% had other various languages. The primary responsibility for managing financial issues was caregivers in 89.16%. Only 7.5% had some discussion with their physicians about their estimated cost before starting treatment (Table 1).

More than two-thirds of male patients (69.2%) and male caregivers (62.2%) knew about the site of their disease site but less than one-third of female patients (30.8%) and female caregivers (37.8%) knew about their site of disease. Nepali as the primary language is correlated with better knowledge of disease status, among both patients and caregivers but was statistically significant only in knowing prognosis among native Nepali speaker caregivers (*p* < 0.001). 62.5% of patients were not even literate and only 6.66% of caregivers had education above bachelors’ level. Better education was correlated with a better understanding for both patients and caregivers, statistically significant (*p* < 0.001) in patients in knowing site, stage and prognosis of disease but only significant (*p* < 0.001) in caregivers in knowing the site of the disease (Table 2).

### Results of qualitative part

The in-depth interviews with three patients provided valuable insights into their experiences and understanding of their medical conditions. Two of the patients were female, diagnosed with breast and cervical cancer, respectively, while the third patient was a male with head and neck cancer.

All three patients knew the site of their disease, but only the patient with breast cancer was aware of the stage of the disease. However, even with knowledge of their diagnosis, none of the patients were aware of their disease prognosis, treatment plan or the estimated cost of their treatment. The narration of all patients is presented based on the major themes (physician’s behaviours, misunderstanding and language barrier) all related to inadequate understanding of the relevant issues and communication.

## Physician’s behaviours

*A few days after attending hospital, the doctor told me that I had cancer. The doctor was quite harsh in her words. On the same day, the doctor gave me a few injections of medicine, which were fairly expensive, and didn't even tell me the purpose of the medicines. Due to the anxiety, I could not give credence to what I had heard in the hospital. We didn't like the behavior of the doctor, and went to a hospital in India for treatment. I had a disease recurrence 6 months post treatment. The doctors in India are very professional and have promised me that they will cure the disease. I don’t know the exact amount spent on my treatment till now, and I'm unaware of the estimated cost of the treatment. As I was always a housewife, my husband and son will pay for my treatment (* ***Ca Breast, female*** *).*

## Misunderstanding

*I know there is some lump in my throat for which treatment is being done. ‘Sekai’ (literal meaning- giving heat in the Nepali language) is being planned for the lump, then I will be disease free. I will buy my own steam inhaler at home, so that I don’t have to come to the hospital for ‘Sekai’. I don't know about my estimated cost but I am sure I can afford a steam inhaler. I will travel back tomorrow to my home and will start ‘Sekai’ at home (* ***Head and neck ca, male*** *).*

## Language barrier

*I am illiterate and speak the Newari language at home. I do not know my disease; the doctor should know it. They have studied the disease, I never attended school, how should you expect me to know about the diagnosis? I came here and had surgery. They took out my uterus and I am alright now. My family knows about the cost of treatment. I only know we have financial difficulty at home, my son is jobless and it is very hard for them to pay treatment costs. I have heard that despite a very expensive treatment, this type of disease(cancer) is never going to be cured (* ***Ca Cervix, female*** *: Translated to the Nepali language by the patient’s caretaker).*

## Discussion

This is the first study of this kind to explore patients’ and caregivers’ understanding of disease status and estimated cost of treatment in Nepalese tertiary cancer setting. Level of understanding about the condition potentially impacts on quality of care and treatment adherence and finally treatment outcome. Basic knowledge about the disease and the related financial aspects can enable patients and caregivers to choose appropriate options of care. This study reveals that the patients and caregivers have inadequate knowledge regarding the site, stage and prognosis of disease and about the total estimated cost of treatment. Our findings are consistent with various previous studies [14–16]. In our study patients’ and caregivers’ knowledge is affected by the level of education, gender and primary language. Better education was associated with a better understanding of disease status according to our study. Likewise, there was a trend toward better knowledge among male patients as they are better educated here. More than the patient, the patient’s caretaker and the family know the disease status better because they bear the primary responsibility for the finances.

As healthcare costs for cancer are higher than other conditions, discussions regarding expenses are relevant and necessary to allow timely interventions that reduce the risk of financial burden [7]. Unlike western countries where they have good insurance coverage for chronic diseases, in our population, there is almost none. Patients with cancer who have the financial means also frequently travel to foreign countries to seek cancer care [17]. Although there are a good number of trained oncologists and specialised cancer care hospitals in Nepal now, still a significant number of Nepalese patients visit foreign countries for cancer management. Many patients spend huge amounts of hard-earned money aboard not knowing their disease prognosis and treatment options available in the country. The phenomenon not only exacerbates the distress for patients and their families in relation to finances, travel time and logistical arrangements in foreign countries but also results in a significant outflow of funds from our country’s reserve. Particularly in our society, there is a saying that cancer is the type of disease that not only takes lives but also causes loss of money and assets. The generalising statement is not true for many curative cases, as the treatment cost is more or less fixed for different cancer sites which ranges from around 1.5 to 20 lakhs (near about 1,200$–15,000$) depending on the sites in a public cancer centre.

As evident from the in-depth interviews as well as the questionnaire survey, there was inadequate, inappropriate and ineffective communication between the patients and the physicians. Patients misunderstood therapeutic radiation to be the use of common heat, both could be referred to by the term ‘*Sekai*’. In order to improve the understanding of the health problem they are fighting, the underlying factors for effective communication between providers and users need to be understood and addressed. Since doctors are expected to provide accurate diagnoses for patients, doctors largely focus on clearly communicating the results of diagnoses to the patients, rather than emotionally empathising with them [18]. In addition to this, usually, multiple doctors are posted in a single room in public hospitals, and multiple patients and families communicating with those doctors make a chaotic environment which again affects comprehension. Patients' belief system significantly influences their comprehension. Within our culture, when a patient receives a cancer diagnosis, there is an immediate influx of relatives and neighbours who share anecdotes about individuals they know with cancer. These stories often contain fabricated elements and are used to project their perceptions of cancer onto those who are newly diagnosed. Because of the belief system that cancer is a non-curative disease, many curative patients do not start or complete their planned treatment. Additionally, a category of patients considers their fate as the cause of cancer and remain apathetic towards their treatment plan.

To address the issues related to low levels of understanding, respective administrative and regulatory agencies need to adopt appropriate policy strategies. At the health facility level, improving communication between service providers (oncologists, physicians and nurses) and service users (patients and caregivers) can improve the understanding about the disease status, prognosis and estimated cost. Additional care needs to be given to enhance health system’s capacity to decrease the workload of the service providers and have ample space. This can help by providing adequate time and peaceful space for good communication. In Nepal, there are 131 official languages according to the Language Commission report 2078/079 [19]. It is very tough to be inclusive to all language speakers. Many healthcare users reported undertaking prior preparation, such as consulting the dictionary or the internet, to gain knowledge about their condition, possible treatment and correct terminology [20]. Local language translators or diverse linguistic resources should be available to assist with translations when needed. To address the issues behind financial difficulty, increasing governmental financial scheme dimensions or promoting private sector insurance coverage can be strategic alternatives. Likewise, at the community level, health literacy or health awareness campaigns are needed.

Provision of the cancer-related counselling desk and provision of coordination section dealing with social and psychological issues may further enhance the comprehension of patients and caregivers.

These kinds of changes are needed to enhance the professionalism in the institutions, to foster trust among the institution and service providers and to improve the level of understanding of the users. This further leads to the enhancement of providers’ capabilities to make shared decisions on healthcare plans, use of needed proper health service on time and to manage scarce financial resources appropriately, and subsequently have improved treatment compliance and outcome.

Our study has some limitations. It is a single-centre cross-sectional study with a small sample. In-depth interview was conducted with only three patients from three major departments of hospital, because of limited time and considering them as representatives of the departments. Most of the patients had a combined modality of treatment and hence differences in understanding based on department could not be evaluated. We did not evaluate understanding in relation to age because most patients being in the middle-aged group. We have not assessed the association of other variables like period of diagnosis and type of health problems with understanding of disease. Details associated with the financial status of patients and caregivers’ socio-economic status or income source are missing in our study. Further study can shed more light on the issues in future.

## Conclusion

Knowledge about the disease's site, stage, prognosis and estimated cost is suboptimal in our study. This can hinder the effective treatment adherence and thereby leads to the poor health outcomes for the service users. Effective communication among service providers and service users may help to improve the level of understanding. Respective institutions and administrations should implement appropriate strategies that can mitigate the barriers and foster the facilitators to effective communication. Further extensive research is needed to gain a comprehensive understanding of the issues in our society*.*

## Conflicts of interest

No financial associations are relevant to the submitted manuscript.

## Funding

This research received no grant from any funding agency in the public, commercial or not-for-profit sectors.

## Author contributions

Concept and study design: Dr Shweta Baral, Dr Sudhir Raj Silwal, Dr Deep Lamichhane, Dr Abish Adhikari and Nancy Bhattarai.

Data analysis and interpretation: Dr Shweta Baral, Dr Sudhir Raj Silwal, Dr Deep Lamichhane, Dr Abish Adhikari and Nancy Bhattarai.

Manuscript draft: Dr Shweta Baral, Dr Sudhir Raj Silwal, Dr Deep Lamichhane, Dr Abish Adhikari and Nancy Bhattarai.

Ethical clearance: Dr Shweta Baral, Dr Sudhir Raj Silwal, Dr Deep Lamichhane, Dr Abish Adhikari and Nancy Bhattarai.

Critical revision of the manuscript: Dr Shweta Baral

Final approval of the manuscript: Dr Shweta Baral, Dr Sudhir Raj Silwal, Dr Deep Lamichhane, Dr Abish Adhikari and Nancy Bhattarai.

## Figures and Tables

**Table 1.** Participants’ characteristics.

Characteristics	Number (%)
Age group of patients	
13–40 years	25 (20.83%)
41–70 years	66 (55%)
71 years and above	29 (24.16%)
Age group of patients	
18–40 years	67 (55.83%)
41–70 years	46 (38.33%)
71 years and above	7 (5.83%)
Sex of patients	
Male	47 (39.16%)
Female	73 (60.83%)
Sex of caregivers	
Male	58 (48.33%)
Female	62 (51.66%)
Primary language at home/work	
Nepali	98 (81.6%)
Newari	11 (9.16%)
Others	11 (9.16%)
Education level of patients	
Not literate	75 (62.5%)
Literate	18 (15%)
Grade 12 or less	17 (14%)
Bachelor’s level and above	10 (8.33%)
Education level of caregivers	
Not literate	0
Literate	12 (10%)
Grade 12 or less	100 (83.33%)
Bachelor’s level and above	8 (6.66%)
Primary responsibility for finances	
Patients	13 (10.83%)
Caregivers	107 (89.16%)
Had financial discussion with treating physicians	
Patients	0
Caregivers	9 (7.5%)

**Table 2.** Patients’ and caregivers’ knowledge about disease status and prognosis.

Participants	Site of disease – number (%)		Stage of disease – number (%)		Prognosis – number (%)	
	Recorded	Concordance with data	Recorded	Concordance with data	Recorded	Concordance with data
**Patients**						
	102 (85)	26 (21.6)	31 (25.83)	16 (13.33)	66 (55)	12 (10)
Sex of patients						
Male	45 (37.50)	18 (18.62)	21 (17.5)	11 (9.16)	36 (30)	4 (3.33)
Female	57 (47.50)	8 (7.84)	10 (8.33)	5 (4.16)	30 (25)	8 (6.66)
*p-*value	0.001		1.000		0.103	
Education of patients						
Not literate	70 (58.33)	4 (3.33)	11 (9.16)	0 (0)	39 (32.5)	0 (0)
Literate and above	32 (26.65)	22 (18.32)	20 (16.65)	16 (13.33)	27 (22.49)	12(10)
*p-*value	<0.001		<0.001		<0.001	
Primary language of patients						
Nepali	91 (75.83)	22 (18.33)	26 (21.66)	13 (10.83)	5 (46.66)	11 (9.16)
Others	11 (9.16)	4 (3.33)	5 (4.16)	3 (2.5)	10 (8.33)	1 (0.83)
*p-*value	0.381		1.000		0.675	
**Caregiver**s						
	110 (91.66)	74 (61.66)	74 (61.66)	48 (40)	56 (46.66)	46 (38.33)
Sex of caregivers						
Male	53 (44.16)	46 (38.33)	39 (32.5)	25 (20.83)	30 (25)	23 (19.16)
Female	57 (47.5)	28 (23.33)	35 (29.16)	23 (19.16)	26 (21.66)	23 (19.16)
*p-*value	<0.001		0.885		0.310	
Education of caregivers						
Below bachelors level	102 (85)	66 (55)	67 (55.83)	42 (35)	48 (40)	39 (32.5)
Bachelor level and above	8 (6.66)	8 (6.66)	7 (5.83)	6 (5)	8 (6.66)	7 (5.83)
*p-*value	0.051		0.410		1.000	
Primary language of caregivers						
Nepali	96 (80)	68 (56.66)	64 (53.33)	41 (34.16)	46 (38.33)	43 (35.83)
Others	14 (11.66)	6 (5)	10 (8.33)	7 (5.83)	10 (8.33)	3 (2.5)
*p-*value	0.064		1.000		0.001	
